# Nutritional Value Addition of Bread, Pasta, and Noodles by Incorporating Leaves of Moringa oleifera

**DOI:** 10.7759/cureus.75793

**Published:** 2024-12-16

**Authors:** Shifat Fatima, Minhaj A Usmani, Arvind K Srivastava

**Affiliations:** 1 Food and Nutrition, Era University, Lucknow, IND; 2 Food and Nutrition, Era's Lucknow Medical College and Hospital, Lucknow, IND

**Keywords:** bioactive compounds, functional food development, moringa oleifera-enriched foods, nutritional enhancement, sensory evaluation, value-added products

## Abstract

Background: The growing demand for natural, health-promoting food products has led to increased interest in integrating nutrient-rich ingredients into everyday foods. The addition of *Moringa oleifera* leaves may increase nutrient profile, including essential amino acids, antioxidants, vitamins, and minerals in edible products.

Aim: The study aimed to optimize the addition of *M. oleifera *leaves in bread, noodles, and pasta and evaluate sensory attributes using a nine-point hedonic scale and nutritional analysis.

Results: The incorporation of *M. oleifera* leaves imparted a distinctive green coloration and speckling to the products. Sensory evaluations indicated that moderate enrichment (5.0% in bread, 7.5% in noodles, and 7.0% in pasta) generally enhanced sensory attributes. Nutritional analysis revealed significant improvement in the nutritional profiles of all the three products having *M. oleifera* leaves. The addition of 5.0% *M. oleifera *leaves in bread increased % levels of energy (carbohydrates, protein, fat, ash, iron, calcium, and vitamin C). Noodles with 7.5%* Moringa* exhibited higher carbohydrates, protein, crude fiber, iron, and calcium, whereas pasta with 7.0% *Moringa* showed increased carbohydrates, protein, and crude fiber but decreased energy and ash content.

Conclusion: Moderate addition of *Moringa* leaves is optimal for maintaining sensory quality and overall acceptability, while excessive concentrations may negatively impact sensory attributes.

## Introduction

In recent years, there has been a growing demand for natural and health-promoting food products, driven by increasing consumer awareness regarding the benefits of functional foods. This shift toward natural ingredients is largely fueled by the desire to enhance overall health, prevent diseases, and utilize plant nutritional wealth. *Moringa oleifera*, often called the "miracle tree," has garnered significant attention due to its impressive nutrient profile and therapeutic properties. *M. oleifera *leaves are particularly noted for their high content of vitamins, minerals, and bioactive compounds, contributing to their wide array of health benefits [1,2].

*M. oleifera* leaves are rich in essential amino acids, antioxidants, and micronutrients such as vitamin C, beta-carotene, calcium, potassium, and iron [1]. These components are crucial in enhancing immune function, reducing inflammation, and combating oxidative stress [3,4]. The leaves also contain significant amounts of iron, which is essential for the production of hemoglobin and preventing anemia [5-7]. The high fiber content in *Moringa* leaves aids in digestion, helps maintain healthy blood sugar levels, and supports weight management by promoting a feeling of fullness [8]. Research has demonstrated the therapeutic potential of *M. oleifera* leaves in managing conditions such as diabetes, hypertension, and hyperlipidemia [9,10,11].

The need for this study arises from the growing consumer demand for natural, health-promoting food products, which may address nutritional deficiencies and promote overall well-being. *M. oleifera*, known for its exceptional nutrient profile and therapeutic properties, offers a promising solution to these demands. By incorporating *Moringa* leaves into widely consumed food items such as bread, noodles, and pasta, this study aims to develop value-added products that not only meet the taste and quality expectations of consumers but also provide substantial health benefits.

## Materials and methods

*Moringa* leaves were collected from nearby areas of Era University, Lucknow, India. The leaves were washed with tap water to remove any dirt or impurities. Subsequently, these were shade-dried for two to three days, and the dried ones were ground into fine powder using a mechanical grinder. The powder was stored in a screw-capped air-tight container until used, The other materials used for making bread, noodles, and pasta, i.e., whole wheat flour, refined wheat flour, yeast, honey, buttermilk, refined sugar, and refined oil were purchased from the local market. 

Preparation of *M. oleifera*-enriched bread, noodles, and pasta

The typical compositions of ingredients for making wheat bread, noodles, and pasta are shown in Table 1. Varying amounts of the crude powder of *M. oleifera* were added, and the bread incorporating 5.0%, 10.0%, and 15.0% were termed T1, T2, and T3, respectively. Whole wheat flour, yeast, salt, sugar, and water were added accordingly to maintain the consistency and rise of the bread dough. After mixing the ingredients thoroughly and allowing the dough to ferment, it was kneaded, shaped, proofed, and finally baked to prepare the *Moringa*-enriched bread. The preparation process of noodles involved mixing the whole wheat flour and *Moringa* leaves powder 2.5%, 5.0%, and 7.5% thoroughly, adding water to form a dough, kneading the dough until smooth, rolling it out, cutting it into noodle shapes, and then drying or cooking the noodles as desired. The preparation process of pasta involved thoroughly mixing the whole wheat flour, refined wheat flour, and *Moringa* leaves powder 5.0%, 7.0%, and 9.0% adding water to form a dough, kneading the dough until smooth, rolling it out, cutting it into pasta shapes, and then drying or cooking the pasta as desired.

**Table 1:** Composition and experimental formulation of Moringa leaves bread, noodles, and pasta

Ingredients	Bread	Noodles	Pasta
Whole wheat flour	250 g	100 g	50 g
Refined flour	-	-	50 g
Instant yeast	2g	-	-
Honey	10g	-	-
Buttermilk	70ml	-	-
Sugar	2g	-	-
Oil	5g	-	-
Water	15 ml	90 ml	90ml
Moringa powder	05, 10, and 15 g	2.5, 5.0, and 7.5 g	5.0, 7.0, and 9.0 g

Sensory evaluation

A comprehensive organoleptic or sensory evaluation was conducted to assess the prepared products' sensory attributes. This evaluation involved a panel consisting of 10 trained members, 10 semi-trained members, and 10 laypersons, ensuring a diverse range of feedback. The sensory attributes were evaluated using a nine-point hedonic scale, a widely recognized method in sensory science for measuring the degree of liking for various parameters [12]. The specific attributes assessed included appearance, color, flavor, taste, texture, and overall acceptability.

Each panelist independently rated the sensory attributes of the products. The nine-point hedonic scale ranged from 1 (dislike extremely) to 9 (like extremely). This method allowed for a detailed and quantitative analysis of the sensory qualities of the *Moringa*-enriched products, providing valuable insights into their acceptability and potential areas for improvement. The sensory evaluation results were systematically recorded and analyzed to determine the overall sensory profile of each product variant, facilitating a comprehensive understanding of consumer preferences and product quality.

Nutritional analysis

The nutritional analysis of the three formulated products incorporating *M.* *oleifera* leaves (bread, noodles, and pasta) was conducted using standardized methods outlined by the Association of Official Analytical Chemists (AOAC). The analysis included the determination of moisture, ash, crude fiber, carbohydrate, protein, fat, vitamins, and mineral content. Each product was first homogenized to ensure uniformity, and then samples were subjected to various AOAC methods: moisture content was measured by oven drying, ash content by incineration, crude fiber by acid-base digestion, carbohydrate content by difference, protein content by the Kjeldahl method, and fat content by Soxhlet extraction. Vitamins and minerals were analyzed using high-performance liquid chromatography (HPLC) and atomic absorption spectroscopy (AAS), respectively. These analyses provided comprehensive data on the nutritional profile of the products, highlighting the impact of* Moringa* leaf incorporation on their overall nutritional value.

**Figure 1:**
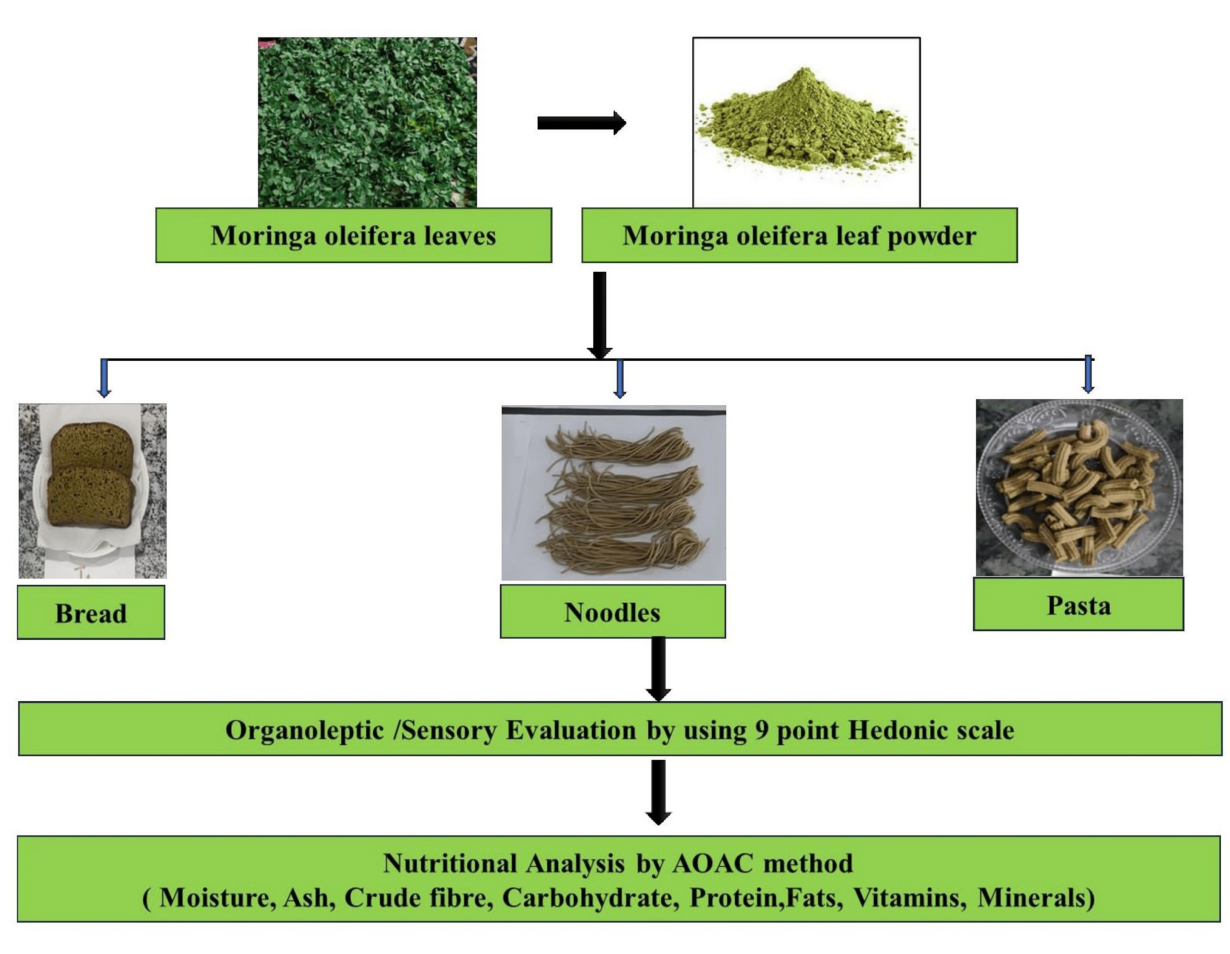
Methodology flow chart: incorporation and evaluation of Moringa oleifera leaves in functional foods

The methodology of the study is summarized in the flowchart (Figure 1), illustrating the preparation, sensory evaluation, and nutritional analysis of *M. oleifera*-enriched bread, noodles, and pasta.

Statistical analysis

Descriptive statistics, i.e., mean and standard deviation (SD), was used to summarize the sensory evaluation scores and nutritional composition, followed by an analysis of variance (ANOVA) to determine significant differences among products with varying concentrations of *M. oleifera* leaves. The p-values were calculated to assess the statistical significance of these differences. Post-hoc analysis was also conducted where necessary to identify specific group differences. The findings were interpreted in terms of both statistical and practical significance, highlighting the impact of *Moringa* incorporation levels on the sensory and nutritional attributes of the products.

## Results

Physical appearance of the formulated products

**Figure 2:**
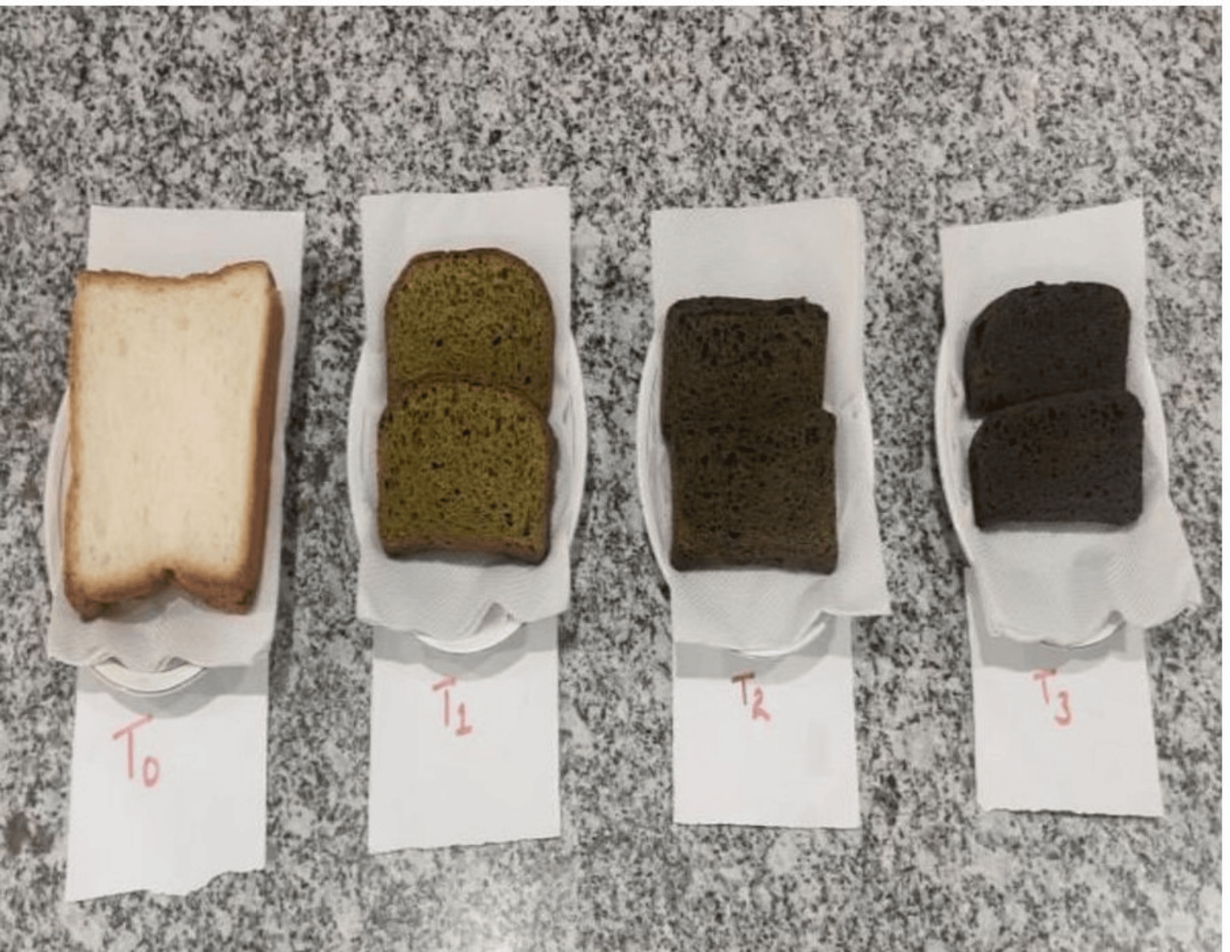
Image of the prepared Moringa bread

**Figure 3:**
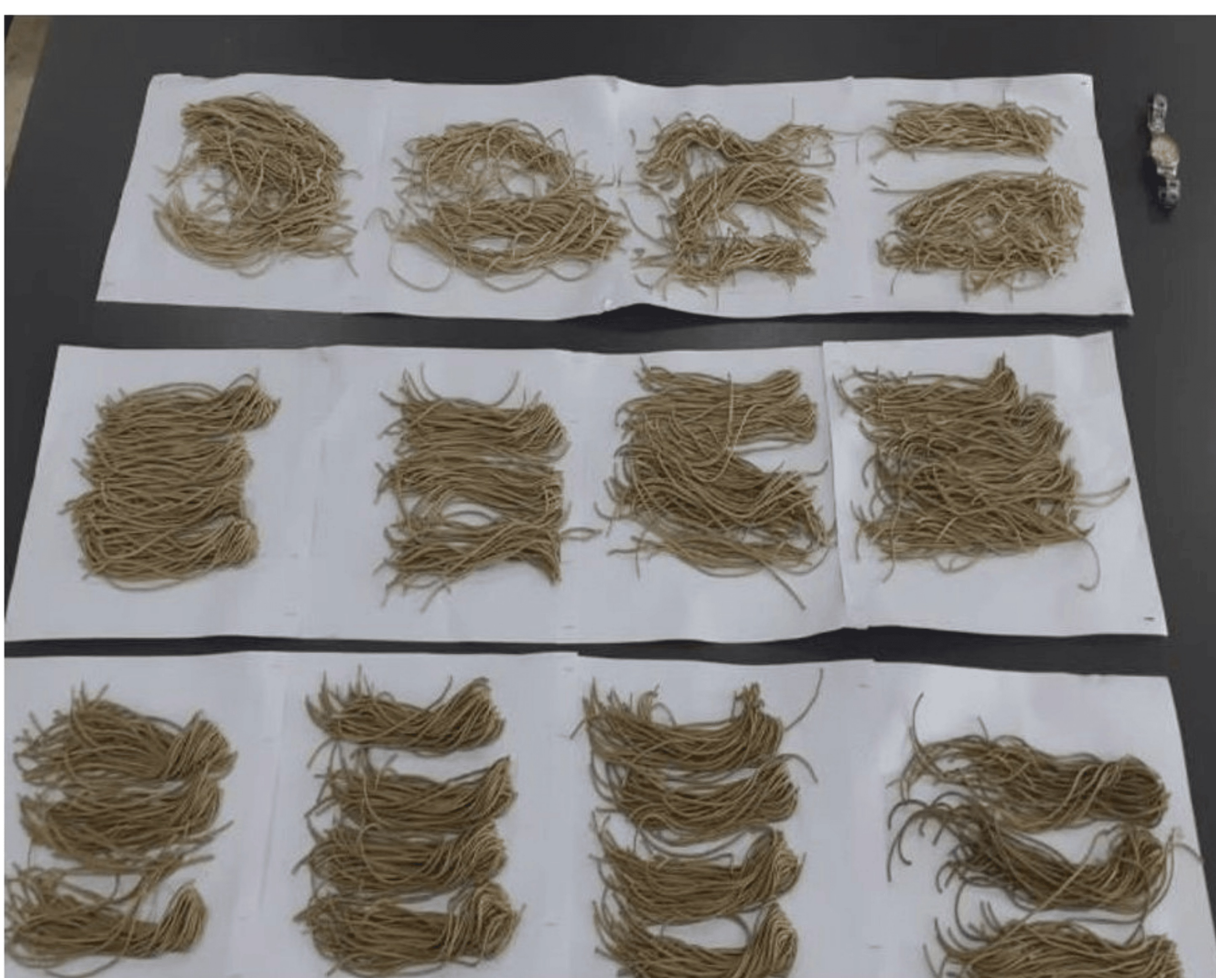
Image of the prepared Moringa noodles

**Figure 4:**
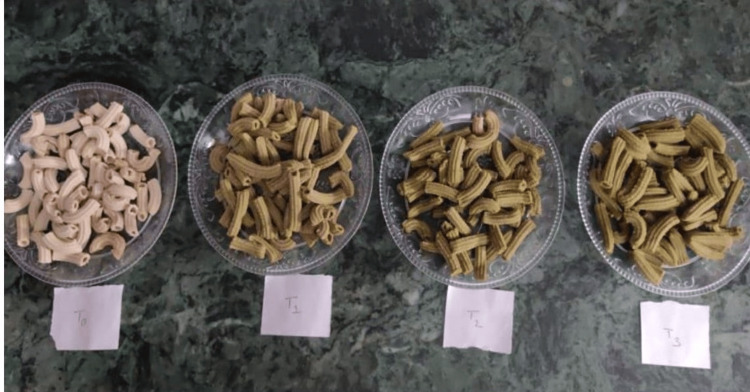
Image of the prepared Moringa pasta

The physical appearance of the prepared products varied significantly with the incorporation of *M. oleifera* leaves. Bread (Figure 2) showed a greenish hue due to the visible presence of fine powder of *Moringa* leaves, enhancing their visual appeal. Noodles (Figure 3) and pasta (Figure 4) showed slight color variations and speckling, which intensified with higher leaf concentrations, imparting a more vibrant appearance. Overall, the products maintained their typical forms and textures, while the addition of *M. oleifera* leaves added a distinct green coloration and speckling effect, enhancing their visual appeal.

Sensory evaluation

Product 1: Moringa Bread

Table 2 presents the organoleptic (sensory) evaluation of* Moringa* leaves-enriched bread in terms of five sensory parameters: color, flavor, taste, texture, and overall acceptability. Four different formulations were tested: T0 (control, no *Moringa*), T1 (moderate *Moringa* enrichment), T2 (higher *Moringa* enrichment), and T3 (highest *Moringa* enrichment).

**Table 2:** Organoleptic evaluation of Moringa bread

Variation	Sensory attributes (Mean ± SD )				
	Color	Flavor	Taste	Texture	Overall acceptability
T0 (0%)	7.75 ± 0.64	8.05 ± 0.76	8.4 ± 0.50	8.05 ± 0.60	8.6 ± 0.50
T1 (5%)	7.65 ± 0.74	7.85 ± 0.67	8.15 ± 0.37	7.65 ± 0.59	7.6 ± 0.68
T2 (10%)	7.1 ± 0.72	6.7 ± 1.03	6.65 ± 0.67	6.55 ± 0.51	7.1 ± 0.64
T3 (15%)	5.7 ± 0.57	4.9 ± 1.37	5.65 ± 0.67	5.9 ± 0.72	6.3 ± 0.66

The results indicate that T1, with moderate *Moringa* enrichment, received the highest scores across all parameters: color (7.75 ± 0.64), flavor (8.05 ± 0.76), taste (8.4 ± 0.50), texture (8.05 ± 0.60), and overall acceptability (8.6 ± 0.50), suggesting that it was the most preferred formulation. The control sample, T0, also performed well but slightly lower than T1 in most parameters. On the other hand, T2 and T3, which had higher levels of Moringa enrichment, received progressively lower scores. T3, in particular, had the lowest scores for flavor (4.9 ± 1.37) and overall acceptability (6.3 ± 0.66), indicating that excessive *Moringa* enrichment negatively impacts the sensory attributes of the bread. Overall, moderate *Moringa* enrichment (T1) appears to strike a balance between nutritional enhancement and sensory quality.

Product 2: Moringa Noodles

Table 3 presents the organoleptic or sensory evaluation of Moringa Noodles, whereas the addition of varying amounts of (T0, T1, T2, and T3) were assessed for their sensory attributes i.e. color, flavor, taste, texture, and overall acceptability. Each value represents the mean ± standard deviation (SD) score given by a panel of evaluators.

**Table 3:** Organoleptic evaluation of Moringa noodles

Variation	Sensory attributes (Mean ± SD )				
	Color	Flavor	Taste	Texture	Overall acceptability
T0 (0%)	7.6 ± 0.88	7.55 ± 0.76	7.25 ± 0.85	7.65 ± 0.81	7.8 ± 0.69
T1 (2.5%)	7± 0.65	7.15 ± 0.74	6.95 ± 0.82	66.8 ± 0.77	6.9 ± 0.85
T2 (5.0%)	6.3 ± 0.86	6.2 ± 0.83	5.9 ± 0.96	6.1 ± 0.78	6.5 ± 0.94
T3 (7.5%)	8.1 ± 0.55	8.3 ± 0.57	8.45 ± 0.51	8.2 ± 0.69	8.6 ± 0.50

T0, the control or standard formulation, shows moderate scores across all attributes, with overall acceptability at 7.8 ± 0.69. T1 shows a slight decrease in scores compared to T0, with overall acceptability rated at 6.9 ± 0.85. T2 shows the lowest scores among all the treatments for all attributes, with overall acceptability at 6.5 ± 0.94, indicating a less favorable sensory profile. By contrast, T3, which likely represents a different formulation or a higher concentration of *Moringa* leaves, received the highest scores for all sensory attributes, with the highest overall acceptability at 8.6 ± 0.50. This indicates that T3 is the most preferred formulation regarding color, flavor, taste, texture, and overall acceptability. The differences in scores suggest varying acceptability and preference for the different formulations of Moringa-enriched noodles.

Product 3: Moringa Pasta

Table 4 presents the results of an organoleptic (sensory) evaluation of *Moringa*-enriched pasta, assessing various sensory attributes including color, flavor, taste, texture, and overall acceptability. The evaluations are categorized into four different samples, labeled T0 through T3. Each sample is rated on a scale, with higher values indicating better sensory attributes. For instance, T2 consistently received the highest scores across all attributes, with color rated at 8.05, flavor at 8.25, taste at 8.45, texture at 8.15, and overall acceptability at 8.4. This suggests that T2 is the most favorably perceived sample in terms of sensory quality.

**Table 4:** Organoleptic evaluation of Moringa pasta

Variation	Sensory attributes (Mean ± SD )				
	Color	Flavor	Taste	Texture	Overall acceptability
T0 (0%)	7.65 ± 0.74	7.45 ± 0.99	7.25 ± 0.71	7.4 ± 0.68	7.2 ± 0.69
T1 (5.0%)	6.95 ± 0.60	7 ± 0.79	7.05 ± 0.76	6.75 ± 0.64	7.1 ± 0.64
T2 (7.0%)	8.05 ± 0.68	8.25 ± 0.64	8.45 ± 0.51	8.15 ± 0.58	8.4 ± 0.59
T3 (9.0 %)	5.9 ± 0.71	5.4 ± 0.99	5.75 ± 0.85	5.8 ± 0.83	5.9 ± 0.78

Conversely, T3 received the lowest scores in most attributes, including color (5.9), flavor (5.4), taste (5.75), and texture (5.8), with an overall acceptability score of 5.9. This indicates that T3 was the least preferred among the samples, showing poorer sensory qualities. T0 and T1 fall between these extremes, with T0 generally receiving slightly better ratings than T1, except for the texture attribute. Overall, the data indicate that T2 is the most acceptable and preferred variant of the *Moringa*-enriched pasta, while T3 is less favored, with T0 and T1 providing intermediate sensory experiences.

Nutritional analysis

Nutrient Analysis of Moringa Bread

Table 5 compares the nutritional content of *Moringa* bread with and without the addition of 5.0% *Moringa* leaves. With the inclusion of *Moringa*, the bread's energy content increases from 260.55 Kcal to 293.22 Kcal. Carbohydrates rise from 53.67 g to 61 g, and protein slightly increases from 8.7 g to 9.2 g. Fat content also increases from 1.23 g to 1.38 g. The ash content, which reflects mineral content, increases from 1.60% to 1.85%, while moisture content decreases from 34.80% to 26.57%. Crude fiber increases from 0.82 g to 1.02 g. Notably, iron content more than doubles from 2.52 mg to 4.62 mg, calcium rises significantly from 151.2 mg to 186.80 mg, and vitamin C content increases markedly from 14.94 mg to 42.85 mg. This demonstrates that adding *Moringa* leaves enhances the bread's nutritional profile, especially in terms of minerals and vitamins.

**Table 5:** Nutrient analysis of Moringa bread

Nutrients	T0 (Control)	T1
Energy (Kcal)	260	293
Carbohydrates (g)	53.6	61.0
Protein (g)	8.70	9.20
Fat (g)	1.23	1.38
Ash (g)	1.60	1.85
Moisture (%)	34.8	26.5
Crude fiber (g)	0.82	1.02
Iron (mg)	2.52	4.62
Calcium (mg)	151	186
Vitamin C (mg)	14.94	42.85

Nutrient Analysis of Moringa Noodles

Table 6 provides a comparison of the nutritional profiles of noodles with and without added *Moringa* leaves. The original noodles (T0) and the noodles with 7.50% *Moringa* leaves (T3) show nearly the same energy content (305 Kcal vs. 306 Kcal) and a slight increase in carbohydrates (75.42 g to 76.42 g) and protein (7.8 g to 8.0 g) with the addition of *Moringa* leaves. Fat content decreases from 0.21 g to 0.09 g, while ash content, representing minerals, increases significantly from 1.01% to 1.75%. Moisture content drops from 12.2% to 9.55%, indicating lower moisture retention with the *Moringa* addition. Crude fiber increases from 3.28 g to 4.19 g, and both iron and calcium contents rise slightly from 4.9 mg to 5.28 mg and 48 mg to 49.11 mg, respectively, reflecting the contribution of *Moringa* leaves to these nutrients.

**Table 6:** Nutrient analysis of Moringa noodles

Nutrients	T0 (Control)	T2
Energy (Kcal)	305	306
Carbohydrates (g)	75.4	76.4
Protein (g)	7.80	8.00
Fat (g)	0.21	0.09
Moisture (%)	1.01	1.75
Crude fiber (g)	12.2	9.55
Iron (mg)	3.28	4.19
Calcium (mg)	4.90	5.28

Nutrient Analysis of Moringa Pasta

Table 7 presents a nutritional analysis of *Moringa* pasta with different *Moringa* concentrations: T0 (the original pasta without *Moringa* leaves), T2 (7%) (pasta with 7.0% *Moringa* leaves added).

**Table 7:** Nutrient analysis of Moringa pasta

Nutrients	T0 (Control)	T2
Energy (Kcal)	274.72	147.99
Carbohydrates (g)	62.13	64.54
Protein (g)	12.2	13
Fat (g)	0.8	0.82
Moisture (%)	1.22	0.47
Crude fiber (g)	22.55	24.06
Iron (mg)	1.10	1.21
Calcium (mg)	3.8	4.24

With 7.0% *Moringa* leaves, the energy content of the pasta decreases significantly from 274.72 Kcal to 147.99 Kcal. Carbohydrates increase slightly from 62.13 g to 64.54 g, while protein increases marginally from 12.2 g to 13 g. Fat content remains nearly the same, with a slight increase from 0.8 g to 0.82 g. Ash content decreases from 1.22% to 0.47%, indicating a lower mineral content compared to the base pasta. Moisture content rises from 22.55% to 24.06%, suggesting that *Moringa* leaves may retain more moisture. Crude fiber content increases slightly from 1.10 g to 1.21 g, and both iron and calcium contents rise slightly with the addition of *Moringa* leaves, reflecting their contribution to these nutrients.

## Discussion

The incorporation of *M. oleifera* leaves into bread, noodles, and pasta introduces physically distinct visual changes. Bread exhibited a greenish hue, enhancing its visual appeal due to the presence of finely chopped *Moringa* leaves. Similarly, noodles and pasta showed color variations and speckling, which became more pronounced with higher *Moringa* concentrations. These observations align with studies like those by Kamble et al. (2022), who noted that *Moringa*'s green pigments significantly affect the appearance of baked products and pasta [13] also align with the findings of Endah et al. (2024) who discussed that the green color of the muffins due to the presence of chlorophyll pigments present in *Moringa *leaves [14]. The green coloration makes the products more attractive and highlights the addition of nutritious ingredients

*Moringa*-enriched bread (T1) received the highest scores in sensory evaluation, such as in color, flavor, taste, texture, and overall acceptability, suggesting a favorable balance between sensory attributes and nutritional enhancement. This is consistent with findings from Bourekoua et al. (2018), who observed that moderate levels of *Moringa *incorporation improved the sensory qualities of baked bread without overwhelming the product's original characteristics [15]. However, higher concentrations (T2 and T3) negatively impacted the sensory attributes, particularly flavor and overall acceptability, aligning with research by Nwakalor et al. (2014) [16], which indicated that excessive *Moringa* leads to stronger, sometimes off-putting flavors that reduce overall acceptability also in line with Milla et al. (2021) [17] and Manivel et al. (2019) [18].

*Moringa* noodles, T3, was rated the highest for all attributes, which contrasts with studies like those by Salha et al. (2023), where higher* Moringa* concentrations were associated with decreased sensory scores [19]. This discrepancy might be due to differences in formulation or sensory panel preferences. In the *Moringa*-enriched noodles, the results indicated that the highest sensory acceptability was at 10.0% *Moringa *enrichment (T2), with flavor, taste, and overall acceptability scoring the highest at this concentration. This is in line with studies by Mpalanziet al. (2023) [20], who reported that 10.0% *Moringa* fortification in pasta products maintained good texture, color, and taste without overpowering the product with bitterness. Similar to our study, both researchers found that sensory scores dropped significantly when *Moringa *levels reached 15.0% or higher, particularly for flavor and texture, which became undesirable to the consumer.

Similarly, T2 pasta received the highest ratings for sensory attributes, while T3 had the lowest, mirroring the findings by Rohman et al. (2024), who reported that higher *Moringa* content can detract from the overall sensory experience [21]. However, the scores for flavor and taste significantly declined at the highest level of fortification (T3, 15.0%), with flavor dropping to 5.4 ± 0.99 and taste to 5.75 ± 0.85. This decline can be attributed to the high phenolic content in* Moringa* leaves, which imparts bitterness when used in excess. Similar results were observed by Coello et al. (2021) [22], who noted a decrease in the palatability of *Moringa*-enriched pasta at concentrations higher than 10.0%.

The incorporation of *Moringa* into bread, noodles, and pasta in the current study demonstrated significant improvements in the nutritional profile of all products, particularly in terms of protein, fiber, vitamins, and minerals. These findings are in line with previous studies that have explored the nutritional potential of *Moringa*-enriched food products, confirming the value of *Moringa* as a functional food ingredient. The nutritional analysis revealed that adding *Moringa* leaves to bread, noodles, and pasta significantly enhances their nutrient profiles. For bread, the addition of *Moringa* leaves increased energy, carbohydrates, protein, fat, and ash content and notably improved levels of iron, calcium, and vitamin C. In noodles, the addition of *Moringa* leaves increased carbohydrates, protein, ash, and crude fiber while decreasing fat and moisture content, supporting research by Kamble et al. (2022), which highlighted the benefits of *Moringa *for enhancing the nutritional profile of pasta products [23]. However, the nutritional analysis of pasta showed a significant drop in energy content with 7% *Moringa* addition, which contrasts with findings from studies like those by Kamble et al. (2022), where *Moringa*-enriched pasta maintained energy content. This drop in energy might be attributed to changes in formulation or ingredient interactions [13].

*Moringa* leaves are known for their high protein content, which was reflected in the increased protein levels across all *Moringa*-enriched products (bread, noodles, and pasta). In this study, higher levels of *Moringa* (T2 and T3 formulations) resulted in significantly elevated protein content compared to the control (T0). This aligns with the finding of Giuberti et al. (2021) [23], who reported that *Moringa* fortification increased the protein content of cereal-based products such as bread and noodles. For instance, the 15% *Moringa*-enriched pasta (T3) in this study showed the highest protein content, consistent with Prayitno et al. (2022), where pasta enriched with 15% *Moringa* had the highest protein increase due to the leaf’s rich amino acid profile [24]. *Moringa* is particularly valued for its high levels of essential amino acids, making it a valuable addition to vegetarian and vegan diets, where plant-based protein sources are crucial.

The fiber content in *Moringa*-enriched products was also significantly higher in the T2 and T3 formulations compared to the control (T0). *Moringa’*s high fiber content contributes to improved digestion and gut health, which is a key health benefit of its incorporation into food products. This observation is supported by Coello et al. (2021) [22] and Eke et al. (2022) [25], who found that *Moringa*-enriched bread and pasta showed an increase in fiber content, which also positively impacted satiety and digestive health in consumers.

Specifically, the 10% *Moringa*-enriched noodles (T2) in this study had notable fiber enhancement, making the product a functional food with potential benefits for managing blood sugar and cholesterol levels. These results are in line with studies like Nudel et al. (2023) [26] and Mehwish et al. (2022) [27], which observed that *Moringa*’s dietary fiber content improved the functional properties of enriched food products while supporting a health-promoting diet.

*Moringa* is a rich source of vitamins (such as vitamins A and C) and minerals (including calcium, potassium, and iron). The fortification of bread, noodles, and pasta with* Moringa* significantly enhanced the micronutrient profile of these products, especially in formulations with higher *Moringa* levels (T2 and T3). For example, the T3 formulation in *Moringa* bread showed a substantial increase in iron and calcium content compared to the control, which is consistent with findings from Mushtaq et al. (2018) [28].

Limitations and restrictions

Despite the positive outcomes, several limitations should be acknowledged. The sensory evaluation relied on a small panel size, which may limit the generalizability of consumer acceptability. Variability in the nutrient composition of *Moringa* leaves due to seasonal and environmental factors could have influenced the findings. In addition, the study primarily focused on short-term evaluations of sensory and nutritional attributes; long-term storage stability, shelf life, and consumer behavior under real-world conditions remain unexplored. Future studies could address these gaps by incorporating larger and more diverse panel sizes and conducting longitudinal studies to assess product stability and acceptance over time.

## Conclusions

Incorporating *M. oleifera* leaves into bread, noodles, and pasta significantly enhances their nutritional profiles by increasing key nutrients such as vitamins, minerals, and protein. Moderate addition of *Moringa* leaves improves the sensory qualities of these products, balancing nutritional benefits with taste and texture, as evidenced by higher sensory scores for moderate formulations. However, excessive *Moringa* enrichment tends to negatively impact sensory attributes, leading to decreased overall acceptability. The findings highlight the potential of *Moringa* leaves as a valuable ingredient for fortifying staple foods, although careful consideration is needed to optimize levels to achieve the best balance between nutrition and sensory appeal.
